# Pre-operative Obesity-Associated Hyperandrogenemia in Women and Hypogonadism in Men Have No Impact on Weight Loss Following Bariatric Surgery

**DOI:** 10.1007/s11695-020-04761-4

**Published:** 2020-06-13

**Authors:** Hannes Beiglböck, Paul Fellinger, Tamara Ranzenberger-Haider, Bianca Itariu, Gerhard Prager, Alexandra Kautzky-Willer, Michael Krebs, Peter Wolf

**Affiliations:** 1 Division of Endocrinology and Metabolism, Department of Internal Medicine III, Medical University of Vienna, Währinger Gürtel 18-20, 1090 Vienna, Austria; 2 Division of Bariatric Surgery, Department of General Surgery, Medical University of Vienna, Währinger Gürtel 18-20, 1090 Vienna, Austria

**Keywords:** Androgen excess, Testosterone deficiency, Functional hypogonadism

## Abstract

**Background:**

In severe obesity, hypogonadism in men and androgen excess in women are frequently observed. Sex hormones play an important role in body composition and glucose and lipid metabolism. However, whether pre-operative gonadal dysfunction impacts weight loss after bariatric surgery is not fully known.

**Methods:**

A total of 49 men and 104 women were included in a retrospective analysis. Anthropometric characteristics, glucose and lipid metabolism, and androgen concentrations were assessed pre-operatively and 17.9 ± 11 or 19.3 ± 12 months post-operatively in men and women. Men with (HYPO_male_) and without (controls: CON_male)_ pre-operative hypogonadism, as well as women with (HYPER_female_) and without (controls: CON_female)_ pre-operative hyperandrogenemia, were compared.

**Results:**

In men, pre-operative hypogonadism was present in 55% and linked to a higher body mass index (BMI): HYPO_male_ 50 ± 6 kg/m^2^ vs. CON_male_ 44 ± 5 kg/m^2^, *p* = 0.001. Bariatric surgery results in comparable changes in BMI in HYPO_male_ and CON_male_ − 16 ± 6 kg/m^2^ vs. − 14 ± 5 kg/m^2^, *p* = 0.30. Weight loss reversed hypogonadism in 93%. In women, androgen excess was present in 22%, independent of pre-operative BMI: CON_female_ 44 ± 7 kg/m^2^ vs. HYPER_female_ 45 ± 7 kg/m^2^, *p* = 0.57. Changes in BMI were comparable in HYPER_female_ and CON_female_ after bariatric surgery − 15 ± 6 kg/m^2^ vs. − 15 ± 5 kg/m^2^, *p* = 0.88. Hyperandrogenemia was reversed in 61%.

**Conclusions:**

Besides being frequently observed, hypogonadism in men and androgen excess in women have no impact on post-surgical improvements in body weight and glucose and lipid metabolism. Weight loss resulted in reversal of hypogonadism in almost all men and of hyperandrogenemia in the majority of women.

## Introduction

Bariatric surgery is an effective treatment option for morbid obesity. Besides favorable effects on body weight, it was shown that cardiovascular risk factors like type 2 diabetes mellitus, hypertension, dyslipidemia, and non-alcoholic fatty liver disease improve following surgery [1, 2], resulting in decreased all-cause mortality [3].

Sex hormones are well known to play an important role in body composition and glucose and lipid metabolism, of which especially androgens are characterized by a sexual dimorphism [4, 5]. In men with hypogonadism, reduced testosterone concentrations are associated with obesity, insulin resistance, and hypertension [6], resulting in increased cardiovascular mortality [7]. Hypogonadism is frequently observed in men with morbid obesity and improves following bariatric surgery [8–10]. Since testosterone deficiency is associated with abdominal obesity and testosterone replacement therapy promotes muscle strength and physical activity, pre-operative hypogonadism might have adverse effects on long-term post-operative improvement in cardiovascular risk factors [11, 12].

In contrast to the favorable impact of testosterone in men, women with hyperandrogenemia are often obese and more likely to have metabolic syndrome. The most common cause of androgen excess in women is polycystic ovary syndrome (PCOS), which is closely linked to insulin resistance and obesity and has an unfavorable influence on women’s quality of life [13–16]. Alterations in sex hormone–binding globulin (SHBG) synthesis in the liver, which is mainly influenced by body fat and insulin, provide one possible mechanism for the increased testosterone levels observed in women with morbid obesity [5, 17, 18]. SHBG binds testosterone with high affinity. Decreased concentrations of SHBG in obesity thus result in a greater percentage of free testosterone and a condition of relative functional hyperandrogenemia [18]. Bariatric surgery might be a promising therapy to improve symptoms as well as insulin sensitivity and hypertension in women with obesity and PCOS [19].

Testosterone plays an important role in the development of metabolic diseases and cardiovascular risk factors in a sex-specific manner. Therefore, this study aims to analyze long-term weight loss and improvements in cardiometabolic risk factors depending on pre-operative androgen hormone status in men and women.

## Patients and Methods

The medical records of all patients in routine care at the obesity outpatients’ clinic of the Medical University of Vienna, Department of Medicine III, Division of Endocrinology and Metabolism, from January 2015 to September 2019 were analyzed retrospectively.

**Fig. 1:**
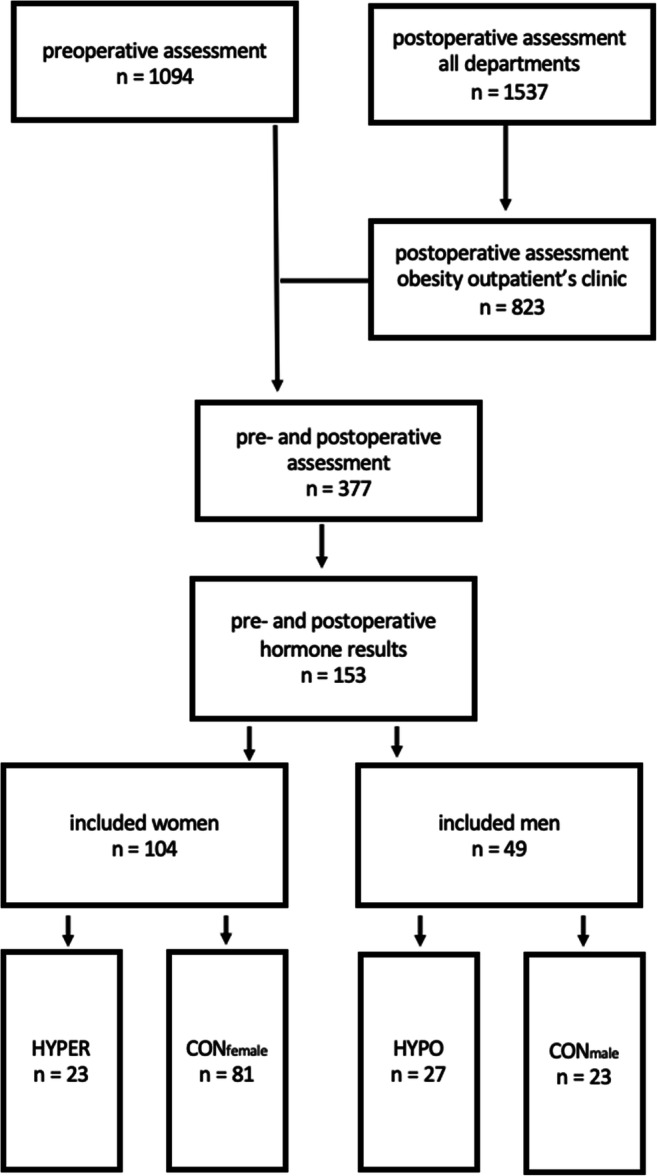
Flowchart of the enrollment process; CON_male_, eugonadal control group; HYPO_male_, presence of hypogonadism before operation; CON_female_, eugonadal control group; HYPER_female_, presence of androgen excess before operation

**Fig. 2:**
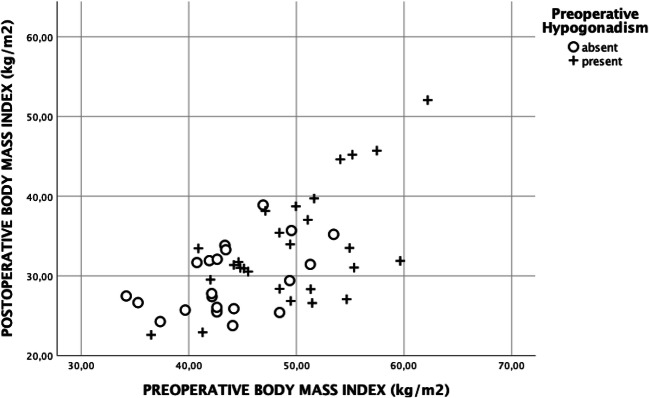
Body mass index before and after bariatric surgery depending on pre-operative gonadal function in men

**Fig. 3:**
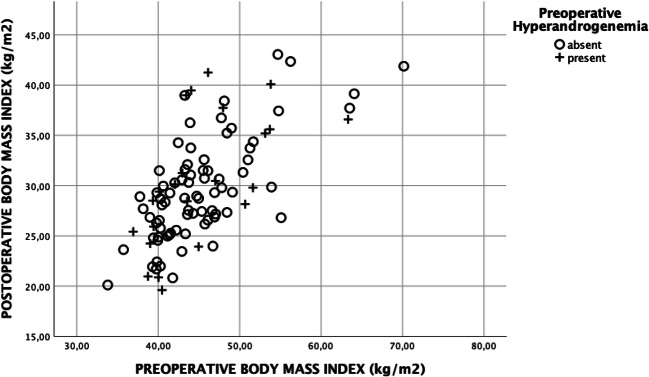
Body mass index before and after bariatric surgery depending on pre-operative gonadal function in women

The study protocol was approved by the ethics committee of the Medical University of Vienna. The inclusion criteria for men and women were history of bariatric surgery for morbid obesity, availability of anthropometric data, and laboratory parameters including androgen concentrations pre-operatively and post-operatively. In total, 49 men and 104 women met these criteria and were included in analysis (see Fig. 1). The mean follow-up period was 17.9 ± 10.9 months for men and 19.3 ± 11.5 months for women. The anthropometric data including height, weight, and body mass index (BMI) were analyzed pre-operatively and at the last follow-up at the obesity outpatients’ clinic (Figs. 2 and 3). The analyzed laboratory parameters included routine chemistry, parameters of glucose and lipid metabolism (glycosylated hemoglobin (HbA1c), triglycerides (TG), total cholesterol, high density cholesterol (HDL)), liver enzymes (gamma-glutamyltransferase (GGT), aspartate aminotransferase (ASAT), alanine aminotransferase (ALAT)), androgens (testosterone, bioavailable testosterone, androstenedione, dehydroepiandrosterone sulfate (DHEAS)), follicle-stimulating hormone (FSH), luteinizing hormone (LH), and sex hormone binding globulin (SHBG).

The conditions of hypogonadism and hyperandrogenemia were defined according to assay-specific reference values (www.kimcl.at). Hypogonadism in men was defined as a testosterone level less than 1.9 ng/mL in men aged 50 years or more and testosterone levels less than 2.5 ng/mL in men aged less than 50 years. To define hyperandrogenemia in women, first FSH levels were used to biochemically define pre- and post-menopausal hormone status. FSH levels greater than 26 mIU/mL were defined as post-menopausal status. In addition, age-dependent values of DHEAS, testosterone, and androstenedione were used for further division. Testosterone levels greater than 0.41 ng/mL in women aged 50 years or more, as well as testosterone levels greater than 0.48 ng/mL in women less than 50 years of age, were considered to be hyperandrogenemia. DHEAS levels greater than 3.4 μg/mL in women 45 years of age or younger, as well as DHEAS levels greater than 2.6 μg/mL in women older than 45 years, were considered as hyperandrogenemia. Cut-off values for androstenedione levels depended on pre- or post-menopausal hormone status. Hyperandrogenemia was defined in post-menopausal women at levels greater than 2.1 ng/mL and in pre-menopausal women at levels greater than 3.4 ng/mL. Hyperandrogenemia was defined by increased concentrations of at least one parameter (DHEAS, testosterone, or androstenedione).

Cushing disease was excluded in all patients in routine care by adequate suppression of morning cortisol concentrations during a 1-mg dexamethasone suppression test pre-operatively performed in routine care.

The men were grouped according to the presence of hypogonadism before bariatric surgery in HYPO_male_ (presence of hypogonadism) or CON_male_ (eugonadal controls). The women were grouped according to the presence of androgen excess before surgery in HYPER_female_ (presence of hyperandrogenemia) or CON_female_ (eugonadal controls). All laboratory parameters were assessed by using routine laboratory methods (www.kimcl.at).

## Statistical Analysis

The statistical analysis was performed using SPSS (IBM, version 25) and Microsoft Excel (Microsoft, 2018). The normal distribution was checked by data visualization. Student *t* tests (paired and unpaired) were used to compare data pre- and post-operatively and to compare the two groups. The data were analyzed using exploratory statistical analysis. The data are given as means ± standard deviations. The statistical significance level was set at *p* < 0.05.

## Results

In total, 153 patients were included. Hypogonadism was present in 27 of 49 men pre-operatively and normalized in 25 patients (93%) after a median follow-up period of 1.49 ± 0.9 years. Body weight and BMI were significantly higher in HYPO_male_ before bariatric surgery (see Table 1). No significant differences in glucose metabolism, triglycerides, and cholesterol were observed pre-operatively. At the last follow-up visit, weight loss and reduction in BMI were comparable between HYPO_male_ and CON_male_. Despite normalization of pre-operative hypogonadism in 25 of 27 patients, levels of testosterone were still significantly higher in CON_male_. Favorable changes in surrogate markers of glucose metabolism and lipid metabolism were observed in both groups, HYPO_male_ and CON_male_. These changes were not significantly different between the groups. Pre- and post-operative data are provided in detail in Table 1. Roux-en-Y gastric bypass (RYGB) was the most frequently performed surgical procedure in men (51%) followed by one anastomosis gastric bypass (OAGB) (29%). Sleeve gastrectomy and single anastomosis duodeno-ileal switch (SADIS) was performed in 14% and 6%, respectively. The frequencies of different bariatric procedures were comparable between CON_male_ and HYPO_male_ (see Table 1).

**Table 1:** Anthropometric data and laboratory parameters in men in the study

	Pre-operative		Follow-up	
	CON_male_	HYPO_male_	CON_male_	HYPO_male_
*N*	22	27	22	27
Follow-up (months)			14.8 ± 9.2	20.5 ± 11.6
Age (years)	41 ± 11	43 ± 11	42 ± 11	45 ± 11
Body weight (kg)	138.7 ± 20.5	154.3 ± 20.7^#^	93.5 ± 17.6*	104.9 ± 22.9*
BMI (kg/m^2^)	43.6 ± 4.8	49.5 ± 6.1^#^	29.3 ± 4.3*	33.6 ± 7.2^#^*
Weight loss (kg)	-	-	− 45.2 ± 14.8	− 49.4 ± 18.5
BMI reduction	-	-	− 14.2 ± 4.7	− 15.9 ± 5.9
RYGB (%; *n*)	50%; 11	52%; 14	-	-
OAGB (%; *n*)	27%; 6	30%; 8	-	-
Sleeve (%; *n*)	18%; 4	11%; 3	-	-
SADIS (%; *n*)	5%; 1	7%; 2	-	-
ASAT (U/L)	33 ± 19	29 ± 10	31 ± 18	26 ± 8
ALAT (U/L)	56 ± 40	46 ± 18	39 ± 24	34 ± 19*
GGT (U/L)	53 ± 49	46 ± 25	38 ± 29	26 ± 15*
TG (mg/dL)	166 ± 80	211 ± 170	94 ± 36*	102 ± 54*
Total cholesterol (mg/dL)	183 ± 42	186 ± 47	145 ± 26*	152 ± 37*
HDL cholesterol (mg/dL)	40 ± 10	41 ± 10	48 ± 15*	52 ± 15*
HbA1c (%)	6.0 ± 1.2	6.1 ± 1.1	5.1 ± 0.3*	5.10 ± 0.5*
Testosterone (ng/mL)	3.47 ± 1.09^#^	1.66 ± 0.40^#^	6.92 ± 2.47*	4.60 ± 1.84^#^*
Bioavailable testosterone (ng/mL)	1.68 ± 0.50^#^	0.97 ± 0.30^#^	2.15 ± 0.57*	1.71 ± 0.49^#^*
DHEAS (μg/mL)	2.28 ± 1.37	2.34 ± 1.23	1.87 ± 1.16*	2.67 ± 1.66
Androstenedione (ng/mL)	1.94 ± 0.85	1.24 ± 0.64^#^	1.56 ± 0.96	1.61 ± 0.84*
LH (mIU/mL)	4.23 ± 1.90	4.34 ± 1.60	6.00 ± 2.47*	5.06 ± 2.17
FSH (mIU/mL)	4.31 ± 2.19	3.92 ± 2.72	5.49 ± 2.42*	5.34 ± 4.64*
SHBG (nmol/L)	32.3 ± 17.2	20.5 ± 9.8^#^	74.5 ± 45.9*	50.5 ± 27.4^#^*
Estradiol (pg/mL)	38.4 ± 13.3	36.0 ± 15.5	32.5 ± 9.9*	36.3 ± 14.2

*CON*_*male*_, eugonadal control group; *HYPO*_*male*_, presence of hypogonadism before bariatric surgery; *p < 0.05 compared with baseline; ^#^*p* < 0.05 compared with CON_male_

In total, 104 women were included in the study, of which 23 fulfilled the criteria of hyperandrogenemia pre-operatively. Androgen excess was resolved in 14 (61%) patients at the follow-up. Body weight and BMI were not significantly different between HYPER_female_ and CON_female_ before surgery (see Table 2). Besides with increased androgen levels, there were no significant differences pre-operatively in glucose metabolism and lipid metabolism between these two groups. Weight loss and reduction in BMI were similar in HYPER_female_ and CON_female_ at the last follow-up. Hyperandrogenemia was still present in nine (39%) women. At the follow-up, the levels of testosterone, DHEAS, and androstenedione in HYPER_female_ were still significantly higher but within the normal range. In both groups at follow-up, favorable changes in lipid metabolism and glucose metabolism were observed compared with baseline (see Table 2). In women, OAGB was performed in 59%, followed by RYGB in 27%. Moreover, sleeve gastrectomy and SADIS was performed in 13% and 2%, respectively. The frequencies of different surgical procedures were comparable between CON_female_ and HYPER_female_ (see Table 2).

**Table 2:** Anthropometric data and laboratory parameters in all included women

	Pre-operative		Follow-up	
	CON_female_	HYPER_female_	CON_female_	HYPER_female_
*n*	81	23	81	23
Follow-up (months)			20.6 ± 11.9	14.3 ± 8.6^#^
Age (years)	40 ± 11	35 ± 10	42 ± 11	36 ± 10
Body weight (kg)	119.7 ± 20.1	123.2 ± 23.6	79.3 ± 14.4*	82.9 ± 19.5*
BMI (kg/m^2^)	44.4 ± 7.1	45.3 ± 6.5	29.4 ± 5.0*	30.5 ± 6.6*
Weight loss (kg)			− 40.4 ± 14.5	− 40.4 ± 17.1
BMI reduction			− 15.0 ± 5.3	− 14.8 ± 5.9
RYGB (%; *n*)	28%; 23	22%; 5	-	-
OAGB (%; *n*)	61%; 49	52%; 12	-	-
Sleeve (%; *n*)	10%; 8	22%; 5	-	-
SADIS (%; *n*)	1%; 1	4%; 1	-	-
ASAT (U/L)	25 ± 12	25 ± 13	24 ± 8	22 ± 5
ALAT (U/L)	33 ± 20	32 ± 18	29 ± 12*	25 ± 11
GGT (U/L)	32 ± 25	29 ± 18	17 ± 16*	15 ± 9*
TG (mg/dL)	137 ± 72	149 ± 60	90 ± 42*	105 ± 43*
Total cholesterol (mg/dL)	182 ± 39	185 ± 40	161 ± 26*	159 ± 33*
HDL cholesterol (mg/dL)	49 ± 13	45 ± 12	59 ± 14*	51 ± 12^#^*
HbA1c (%)	5.7 ± 0.8	5.8 ± 0.7	5.1 ± 0.4*	5.3 ± 0.4*
Testosterone (ng/mL)	0.23 ± 0.11	0.56 ± 0.18^#^	0.19 ± 0.12*	0.39 ± 0.18^#^*
Bioavailable testosterone (ng/mL)	0.09 ± 0.06	0.23 ± 0.10^#^	0.04 ± 0.04*	0.08 ± 0.06^#^*
DHEAS (μg/mL)	1.37 ± 0.66	2.97 ± 1.12^#^	1.16 ± 0.59*	2.32 ± 1.16^#^*
Androstenedione (ng/mL)	1.24 ± 0.67	2.77 ± 1.19^#^	1.14 ± 0.61	2.16 ± 0.98^#^
LH (mIU/mL)	11.52 ± 10.97	10.71 ± 9.30	14.19 ± 14.17*	14.36 ± 16.37
FSH (mIU/mL)	13.02 ± 16.19	10.03 ± 12.58	20.33 ± 25.30*	17.30 ± 25.55
SHBG (nmol/L)	48.9 ± 39.1	44.1 ± 38.0	103.5 ± 51.1*	112.2 ± 66.5*
Estradiol (pg/mL)	64.7 ± 66.3	72.0 ± 61.6	106.4 ± 174.8*	414.5 ± 1561.3

*CON*_*female*_, eugonadal control group; *HYPER*_*female*_, presence of androgen excess before bariatric surgery; *p < 0.05 compared with baseline; ^#^*p* < 0.05 compared with CON_female_

## Discussion

Our study demonstrates that (i) alterations in androgen levels are frequently observed in bariatric surgery candidates and that (ii) the presence of hypogonadism in men and androgen excess in women has no impact on weight loss and the favorable changes in post-operative glucose and lipid metabolism. This study adds to the knowledge of sex-specific changes in androgen levels following bariatric surgery for both women and men.

Obesity has a crucial impact on testosterone levels in both sexes, which might be explained by alterations in the synthesis of SHBG. Sex hormones are lipophilic and therefore bind to SHBG in the blood. Decreased SHBG synthesis in the liver is found in men and women with obesity. A decrease in SHBG levels is temporarily associated with an increase in free testosterone. In men, testosterone is then converted into estradiol by the aromatase located in the adipose tissue. Estradiol itself has a suppressing effect on gonadotropins and therefore results in decreased testosterone levels [17, 20, 21]. Other mechanisms such as alterations in central insulin and leptin signaling might also have an impact on GnRH secretion [22]. Depending on the severity of obesity, different pathophysiological mechanisms are suggested to lower testosterone in men. Decreased SHBG is the predominant factor in men with mild obesity, whereas suppression of the hypothalamic-pituitary-testicular axis by inflammatory cytokines and leptin might be mainly responsible for low testosterone in men with severe obesity [23]. In our analysis, we observed a significantly higher BMI and a significantly lower SHBG in men with low testosterone levels before bariatric surgery compared with those with normal testosterone levels.

The pre-operative prevalence of hypogonadism in our study was 55%, which is comparable with previous reports [24]. Of note, in the follow-up after bariatric surgery, testosterone levels increased significantly and hypogonadism resolved in nearly all men (25/27, 93%). Despite differences in absolute body weight, the amount of weight reduction between HYPO_male_ and CON_male_ was comparable. Based on these observations, one might deduce that pre-operative testosterone substitution to treat hypogonadism in men with morbid obesity is not indicated, since favorable effects on body weight and sex hormones are observed in line with weight loss.

Testosterone as such also modifies muscle and fat mass, as well as insulin sensitivity, highlighting the mutual dependency. Hypogonadism due to therapy with GnRH agonists in men with prostate cancer is associated with increased fat mass [25, 26], whereas testosterone treatment in hypogonadism results in improved insulin sensitivity and decreased fat mass in patients with type 2 diabetes mellitus [27]. Interestingly, there is a large variety in the impact of testosterone on glucose and lipid metabolism depending on sex. Whereas in men anabolic effects of testosterone on muscle mass exert favorable metabolic benefits, hyperandrogenemia in women is associated with a higher risk of cardiovascular disease [4, 5]. Also, fat mass increases following long-term testosterone administration in female-to-male transsexuals [28].

In women, insulin resistance might play a major role in the development of androgen excess by modulation of SHBG concentrations, as well as by direct effects on adrenal androgen secretion [16]. Increased frequencies in GnRH pulses leading to LH excess with subsequent increased androgen production in the ovaries also play a major role in the pathogenesis of hyperandrogenemia, which was reported in PCOS [29]. In addition, changes in the 17beta hydroxysteroid dehydrogenase type 3, which converts androstenedione into testosterone in adipose tissue, might be a substantial factor contributing to hyperandrogenemia [30]. Of note, in our cohort, the presence of androgen excess was independent of body weight and BMI, probably highlighting sex-dependent differences in the pathogenesis of testosterone production.

With regard to pre-operative sex hormone status in women, the prevalence of hyperandrogenemia was 22%. Of note, in contrast to men, BMI did not differ between HYPER_female_ and CON_female_. In addition, all markers of hyperandrogenemia (e.g., testosterone, DHEAS, and androstenedione) were substantially decreased in both groups following bariatric surgery, but still significantly higher in the HYPER_female_ group. Hyperandrogenemia was reversed by bariatric surgery in 61% of women. This decrease in androgen levels observed in our study might partly explain previously published increased fertility rates in women following weight loss after bariatric surgery [31].

The major limitation of our study is the retrospective study design with all its known disadvantages. In addition, the follow-up time between the groups was slightly different. However, the estimated influence on weight loss might not be substantial, as it was shown that the majority of weight loss takes place within the first year after bariatric surgery [32, 33]. The sample size is relatively small, which is in part explained by the limited availability of a total hormone status before and after surgery. Moreover, the number of women included in this study was twice the number of men, which is line with literature and daily clinical practice, since the majority of bariatric surgery candidates are women [34]. The cause of androgen excess in women could not be defined, and no detailed information on menstrual cycle or oligomenorrhea was available in patients’ records. Based on the literature, PCOS might have been the most common differential diagnosis, with an estimated prevalence of about 30% based on previous reports [9]. However, at least Cushing syndrome was excluded by the 1-mg dexamethasone suppression test pre-operatively in all patients. In addition, detailed information on medication potentially interfering with sex hormone levels is missing. Furthermore, insulin levels were not available in most of the patients; thus, investigating the effects of improved insulin sensitivity on androgen concentrations is not possible in our cohort. With regard to differences in bariatric procedures, RYGB was most frequently performed in men, whereas OAGB was more prevalent in women. However, surgical techniques were comparable between HYPO_male_ and CON_male_, as well as between HYPER_female_ and CON_female_. Differences in surgery procedures between men and women therefore do not affect our data, since sex-specific analysis of post-surgical outcome was performed. Further large, prospective studies are warranted to prove the impact of gonadal function on weight loss and vice versa following bariatric surgery.

Taken together, our results suggest that bariatric surgery is a very effective way to reverse obesity-associated hypogonadism in men. In women, androgen excess is observed frequently and independently of pre-operative BMI. Despite decreased androgen concentrations following bariatric surgery, remission rates of hyperandrogenemia are lower compared with men. However, hypogonadism and androgen excess have no impact on post-surgical improvements in body weight and glucose and lipid metabolism in both sexes.
